# Why do authors persist in submitting trial reports that do not meet the journal eligibility criteria or *AllTrials* standards?

**DOI:** 10.1002/nop2.1699

**Published:** 2023-03-13

**Authors:** Jane Noyes

**Affiliations:** ^1^ Health and Social Services Research and Child Health Bangor University Bangor UK

It has been well over 12 months since the Editors in Chief of three Wiley Nursing Journals published a joint Editorial reminding authors of their responsibility to meet journal eligibility criteria for trial reports and the AllTrials standards (AllTrials, n.d.) to which Wiley Journals has signed up (Jackson et al., 2021). The author guidelines were also subsequently updated and linked with two previous Editorials that summarised the journal requirements for prospective registration before the first participant was recruited and outlined the common unacceptable issues picked up by editors concerning trial reports (Noyes, 2018, 2021).

At the beginning of 2022, a dedicated Quantitative Intervention Evaluation (QIE) Editor role was created for Jane Noyes at the Journal of Advanced Nursing (JAN) to handle all new quantitative intervention papers and revise workflows. The role worked well and has been effective at identifying issues with submitted papers. This model was subsequently replicated at the Journal of Clinical Nursing (JCN) and Nursing Open (NOP). The JCN and NOP QIE Editors undertook additional training and orientation over the Summer of 2022. A dedicated group of statistical reviewers was also established to handle these papers across the three journals.

Since the publication of the joint Editorial (Jackson et al., 2021), we have closely monitored submissions, and in this Editorial, we provide an update on our experience with a particular focus on JAN where the new processes have been in place the longest.

Over this time, we have seen little to no meaningful changes in JAN author behaviour to those highlighted in previous Editorials (Jackson et al., 2021; Noyes, 2018, 2021). Many of these issues are also being seen with papers submitted to JCN and NOP. Similar to the issues raised by others (e.g. Burbridge, 2022), concerns persist regarding tactics that some authors continue to use (knowingly or unknowingly) in order to get their trial reports into the publication pipeline. We are disappointed that these highly questionable practices continue. In some cases, we are dealing with quite blatant inaccuracies. In the most serious cases, we have written to the author's institution to share our concerns.

Ongoing author and trial report issues include:

- Trials not being registered on authorised trial registries when they were eligible.
- Authors claiming that their trials were prospectively registered before the first participant was recruited, but the dates in the trial registration entry indicate otherwise.
- Authors reporting the registration number of a totally different trial to that reported in their submission.
- The trial described in the trial report differing (sometimes substantially) from the trial registry entry and/or the protocol with no explanation provided.
- The sample size calculation not undertaken for the primary outcome.
- Selective reporting of outcomes.
- Not reporting all outcomes in a single trial report.
- Manipulation of primary and secondary outcomes to favour outcomes with the biggest effect.
- Inappropriate or poorly conducted analyses.
- The reported trial being under‐powered and the results, conclusions and recommendations for practice not supported by the evidence.
- Submitting manuscripts as quantitative or mixed‐methods studies and avoiding mentioning that the study design was actually a trial, presumably to avoid the requirements of registration.
- Re‐submitting trial reports that have previously been rejected by the journal even though the identified issues were not resolvable (such a retrospective registration)—presumably in the hope that the same issues would not be picked up second time round.
- Various concerns about ethics approvals, including whether the trial had been approved by an ethics committee. In at least one case, investigations revealed that the trial was not approved by an ethics committee.
- Trials that significantly favoured the intervention, and no conflict of interest was stated when the trial was funded by the manufacturer of the intervention.
- Concerns about lack of research governance within the institutions from which the trial reports were submitted.

The aforementioned issues are the precise reasons why the AllTrials Initiative was launched in 2013. Our key message to those submitting trials that do not meet the journal or AllTrials requirements is that these issues will very likely be picked up during the preliminary assessment of the manuscript.

We have found that very carefully assessing each submission in line with the journal eligibility criteria and AllTrials standards and weeding out suspect and flawed papers takes a huge amount of Editor time and effort. A single manuscript can take 1–2 h to undertake the initial Editor checks. This process entails comparing the trial report with the trial registration entry and the protocol (if available). To do this, the documents are lined up, side by side on separate computer screens. Any required clarifications are raised with the authors via email and the response awaited. If manuscripts are rejected, a detailed list of the reasons why is provided along with a request not to resubmit the manuscript unless all the issues can be resolved.

Although it can take some time for new publication workflows and processes to bed in, ongoing monitoring shows that these changes have made a positive difference at JAN where we have monitoring data for a full 12 months. Of around 250 submitted trial reports (some submitted more than once), many concerns have been identified as listed above. Some authors who did not provide all the required information to undertake the initial trial integrity checks at the first submission were invited to resubmit manuscripts if the authors could meet the journal and AllTrials criteria. A system of ongoing monitoring, training and development has also been put in place to ensure greater awareness of emerging quality issues around quantitative intervention papers. As this new process has been embedded in JCN and NOP, we have identified a small number of published manuscripts where we have queries about complete adherence to the journal eligibility criteria and AllTrials standards and are currently in contact with the authors regarding the same.

At present, the onus of undertaking the initial detailed integrity checks lies with the QIE editors. There is, however, universal agreement that the Editor and peer reviewer processes of journals are not primarily designed to detect research misconduct and fraud and no process can be 100% effective (Smith, 2006; UK Government Science and Technology Committee, n.d.; UK Research Integrity Office, n.d.). The longer‐term goal needs to be raising author awareness about research misconduct and fraud and ensuring better compliance with journal eligibility criteria and the requirements of the AllTrials initiative. It is incumbent that institutions undertaking or overseeing trials ensure their staff are subject to robust research regulation and governance standards and procedures and are aware of the selected journal and the AllTrials requirements so that we may see improvements in the near future.
